# Novel Oral CGRP Receptor Antagonist Atogepant in the Prevention of Migraine

**DOI:** 10.15190/d.2023.6

**Published:** 2023-06-30

**Authors:** Selia Chowdhury, Tirth Dave

**Affiliations:** ^1^Clemson University, Clemson, SC 29634, USA.; ^2^Bukovinian State Medical University, Chernivtsi, Ukraine

**Keywords:** Atogepant, CGRP antagonist, migraine, headache.

## Abstract

Advancements in molecular biology and neuroscience have uncovered calcitonin gene-related peptide (CGRP), a neuropeptide consisting of thirty-seven amino acids that plays a crucial role in migraine pathogenesis. CGRP receptor antagonist or gepant is an oral medication that can impede the nociceptive signaling pathway related to CGRP. Atogepant, the latest CGRP antagonist approved by the Food and Drug Administration (FDA) for prophylaxis of episodic migraine, works by non-competitively blocking CGRP receptors, thereby curtailing neurogenic inflammation and pain sensitization. Numerous trials have demonstrated that atogepant is an effective therapy for migraine prevention, with its extended half-life and minimal risks of cardiovascular or liver toxicity making it the first drug in its class primarily authorized for that purpose. In terms of monthly migraine days, monthly headache days, and acute medication usage days, atogepant demonstrated a statistically significant difference from baseline.  It was well-tolerated with low adverse event rates. The most commonly reported adverse events were constipation and nausea. Atogepant appears to be beneficial for migraine prevention, and it may be more useful in those who do not want to take the medication as an injection or who do not require a lengthy duration of pharmacological impact. In this article, we provide a systematic review of the literature on atogepant and migraine, emphasizing current achievements in this field of study.

## 1. Introduction

**Figure 1:**
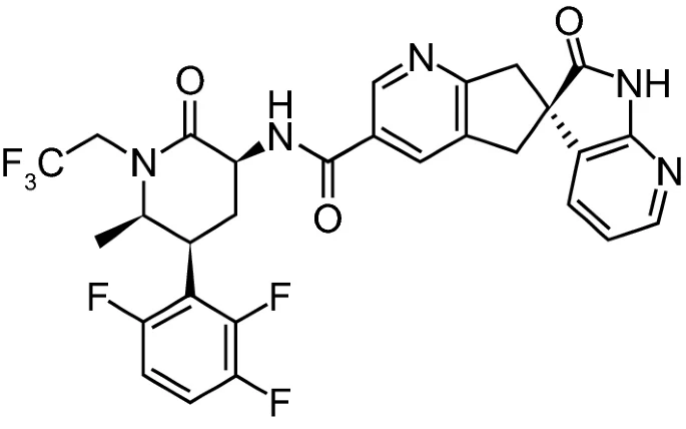
Chemical structure of Atogepant^3^ (reproduced with permission)

Migraine is one of the most common neurological disorders affecting nearly 16% of the population in the United States^1^. It can be a severely debilitating condition, with over 43% of migraine patients reporting moderate-to-severe disability^2^. Migraine is characterized by recurrent headaches lasting 4-72 hours that are usually moderate or severe, pulsating, and unilateral^3^. These headache attacks can be accompanied by nausea, vomiting, and sound/light sensitivity, and can be preceded by an aura (sensory disturbances/physical sensations). They can occur as episodic migraine (≤14 headache days per month) or chronic migraine (> 15 headache days per month and ≥ 8 days with migraine features)^3^. Migraine was once thought to be a vascular disorder, with headache pain associated with vasodilation^4^. Although the pathophysiology of this disorder is not fully understood, advances in migraine research have suggested that neurogenic inflammation and other mechanisms may be triggering the pain^4^. Neuropeptides have been shown to play an important role in pain signaling and modulation in migraine^4^. Calcitonin-gene-related peptide (CGRP), a 37 amino acid neuropeptide, has been linked to migraine pathogenesis. CGRP is a vasodilatory neuropeptide that is released during migraine attacks and has receptors in the human cranial vasculature, trigeminal ganglion, and smooth muscle cells^5^. In recent years, agents that target the CGRP pathway have been developed^3^, ushering in a new era in migraine treatment. Atogepant (Figure 1) an orally administered, small-molecule CGRP receptor antagonist developed for migraine prevention, is one such agent.

**Figure 2:**
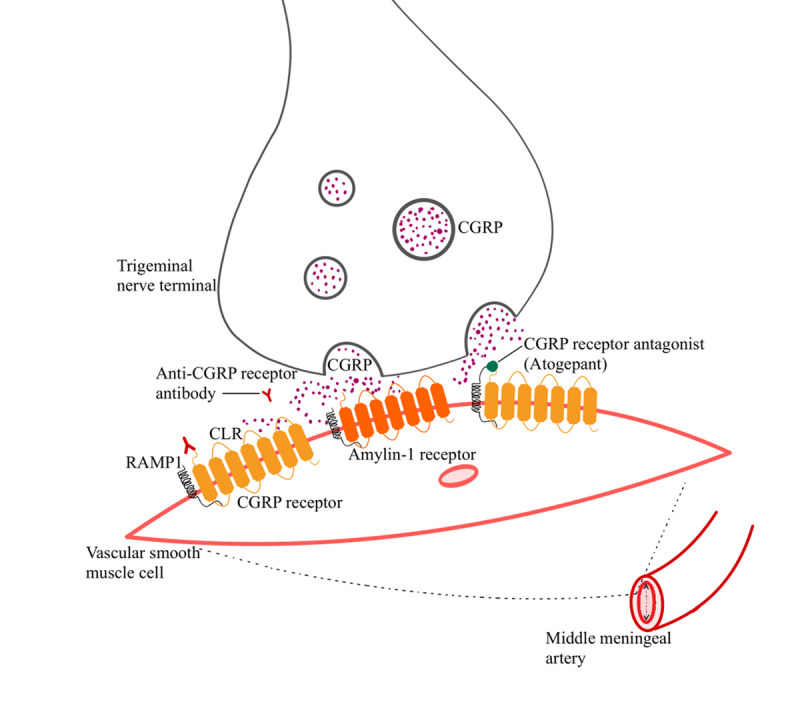
Mechanism of action of anti-CGRP medications

It impedes CGRP receptor signaling by blocking CGRP from binding to and activating this receptor (Figure 2). In CGRP-receptor-expressing cellular models, atogepant inhibits human CGRP-stimulated functional receptor responses (cAMP) with subnanomolar potency. The precise sites of action of atogepant, however, are unknown. The trigeminovascular system's peripheral sites are likely to be crucial^6,7^. Atogepant was approved in the United States on September 28, 2021, for the prevention of episodic migraine in adults^6,8^. The recommended daily dose is 10, 30, or 60 mg, taken without regard for food^6^. In addition, atogepant is in phase 3 clinical development for the prevention of chronic migraine in a number of other countries. It is the first oral CGRP receptor antagonistspecifically developed for migraine prevention^8^.

This systematic review examines the efficacy, tolerability, and safety of a range of oral doses of atogepant, as well as its potential role in migraine therapy.

## 2. Study Selection

A systematic review was conducted to identify randomized controlled trials (RCTs) of atogepant therapy for migraine prophylaxis. From 2018 to January 2023, a systematic search of the National Institute of Health, US National Library of Medicine Clinical Trials, PubMed, European PMC, the Cochrane Library was conducted using the following search terms: atogepant, MK-803, and migraine. Conference abstracts from the Cochrane database as well as drug information from the FDA label were also examined. This review included relevant articles published in English that evaluated the efficacy, safety, and tolerability of atogepant in migraine prevention. Nonprimary literature such as reviews, meta-analyses, and secondary analyses were not included. The conference proceedings were excluded from review because they were from clinical trials, which are discussed in detail.

## 3. Search Results

**Figure 3:**
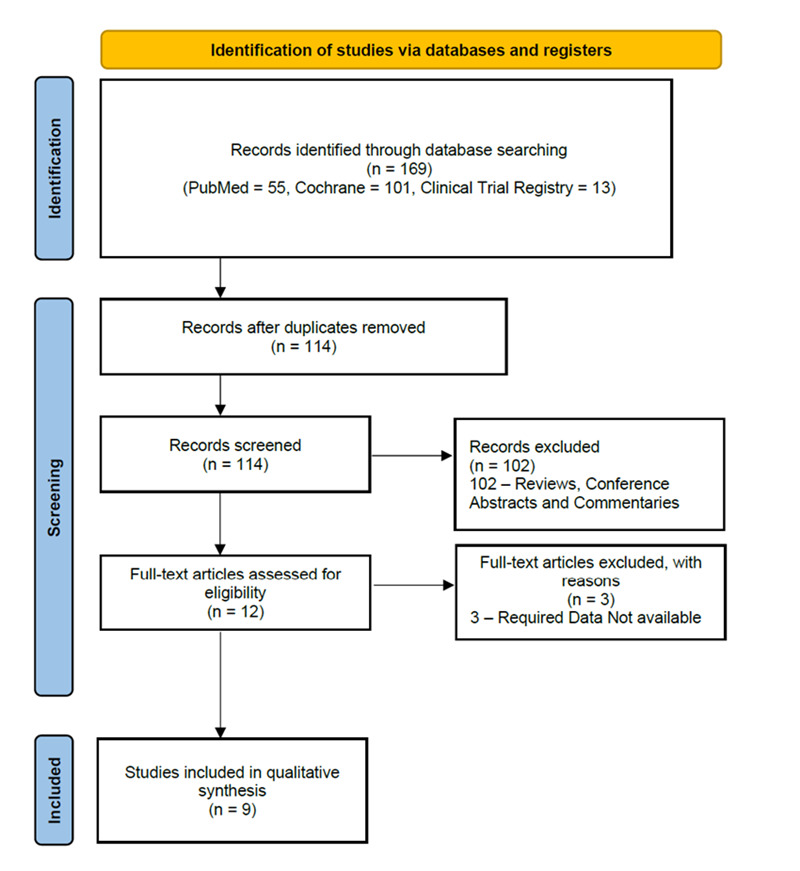
PRISMA flow diagram showing selection process of the studies included in this review

Initially, 169 studies were identified, and 114 studies were retained after duplicates were removed; among these, 9 clinical trials were included for final analysis because they focused on the efficacy, tolerability, and safety of atogepant (Figure 3). There was no research discovered that was not in English.

## 4. Trials and Participant Characteristics

A total of 5139 patients were collectively included in trials selected in this review. Six trials used a double-blind design^9-15^, while the remaining 3 used an open-label design^9,16,17^. Seven trials reported placebo-controlled data^10-15,17^, while the remaining two trials only reported data for atogepant^9,16^. The demographic analysis of the 9 clinical trials included in this review revealed that the majority of the participants were female (78.38%) and white (76.68%). The average age and body mass index (BMI) of patients across all studies were 40.91 (standard deviation: 3.89) and 29.85 (standard deviation: 1.59), respectively. The demographic data is presented in more detail in Table 1.

**Table 1:** Description of Trials and Characteristics of Trial Participants F: Female, M: Male, OD: Once daily, BID: Twice daily, SD: Single Dose, CFB: change from baseline, MMDs: Monthly migraine days, MHDs: Monthly headache days, TRAEs: Treatment-related adverse events, SAEs: Serious adverse events, OR: Odds ratio, CI: Confidence interval; *p <0.05, **p<0.001, vs. placebo; ^a^The data are presented as difference from placebo.

Study	Publication Date	Type of study	Phase	Doses	No. of Participants	Sex	Mean Age (standard deviation)	White (%)	Mean BMI (standard deviation)
Ankrom et al.^9^	April, 2020	Open‐label, 2‐period, fixed‐sequence, single‐site study	I	60 mg OD	26	F: 26 M: 0	56 (5.1)	88.5	26.4 (2.5)
Goadsby et al.^10^	August, 2020	Randomized, double-blind, multicenter clinical trials	II/III	10 mg OD	93	F: 82 M: 11	39·4 (12·4)	74	29.9 (7.3)
				30 mg OD	183	F: 166 M: 17	41·0 (13·6)	79	30.0 (7.1)
				60 mg OD	186	F: 158 M: 28	40·4 (11·7)	72	30.0 (7.8)
				30 mg BID	86	F: 73 M: 13	38·5 (11·2)	85	29.7 (7.2)
				60 mg BID	91	F: 83 M: 8	39·7 (11·9)	78	30.4 (7.4)
				Placebo	186	F: 154 M: 32	40·5 (11·7)	74	30.4 (7.6)
Min et al.^11^	November, 2020	Randomized, double-blind, placebo-controlled	I	170 mg OD	23	F: 4 M:19	37.1 (9.5)	39.1	-
				Placebo	11	F:3 M:8	39.8 (9.3)	54.5	-
Boinpally et al.^12^	May, 2021	Randomized, single‐center, double‐blind, 3‐period, 6‐sequence, single‐dose, crossover trial	I	300 mg SD	60	F: 32 M: 28	33.6 (7.3)	56.7	25.7 (2.9)
				Placebo	59	F: 31 M: 28	33.5 (7.3)	57.6	25.6 (2.9)
Ailani et al.^13^	August, 2021	Randomized, double-blind, multicenter clinical trials	III	10 mg OD	221	F: 200 M: 21	41.4 (12.1)	81.9	30.3 (7.6)
				30 mg OD	228	F: 204 M: 24	42.1 (11.7)	White: 81.1	31.1 (7.6)
				60 mg OD	231	F: 199 M: 32	42.5 (12.4)	White: 83.1	29.9 (7.3)
				Placebo	222	F: 198 M: 24	40.3 (12.8)	White: 87.4	30.8 (8.7)
Schwedt et al.^14^	January, 2022	Randomized, double-blind clinical trial	III	10 mg OD	221	F: 200 M: 21	41.4 (12.1)	White: 81.9	30.4 (7.6)
				30 mg OD	228	F: 204 M: 24	42.1 (11.7)	White: 81.1	31.2 (7.6)
				60 mg OD	231	F: 199 M: 32	42.5 (12.4)	White: 83.1	29.9 (7.3)
				Placebo	222	F: 198 M: 24	40.3 (12.8)	White: 87.4	30.8 (8.7)
Lipton et al.^15^	June, 2022	Randomized, double blind, placebo-controlled clinical trial	III	10 mg OD	221	F:200 M:21	41.4 (12.1)	White : 81.9	30.4 (7.6)
				30 mg OD	228	F:204 M:24	42.1 (11.7)	White : 81.1	31.2 (7.6)
				60 mg OD	231	F:199 M:32	42.5 (12.4)	White : 83.1	29.9 (7.3)
				Placebo	222	F:198 M:24	40.3 (12.8)	White : 87.4	30.8 (8.7)
Klein et al.^16^	January, 2023	Randomized, open-label, multicenter extension trial	III	60 mg OD	685	F:604 M:81	41.8 (12.3)	White : 84.4	30.58 (7.82)
Ashina et al.^17^	January, 2023	Randomized, open-label, multicenter clinical trial	III	60 mg OD	546	F:479 M:64	42.5 (12.0)	76.6	30.6 (8.0)
				Placebo	198	F:172 M:24	41.1 (12.1)	74	30.6 (8.0)

All of the 9 clinical trials included in this review were performed in the United States. The inclusion criteria of five trials specified that patients must have 4 to 14 migraine days per month and onset of migraine before the age of 50^10,13-15,17^. The inclusion criteria for the remaining trials varied, with one study specifically enrolling bilaterally oophorectomized or postmenopausal women^9^. Two studies required that patients did not smoke^11,12^ or use nicotine products in the 2 years prior to the study^12^. The study conducted by Klein et al.^16^ was an extension of the ADVANCE phase 3 trial^10^, thus its participant inclusion criteria were similar to those of the earlier study.

## 5. Efficacy and Safety

The efficacy of atogepant in reducing Monthly migraine days (MMDs) was demonstrated across all studies and doses included in the analysis. The results of the study by Goadsby et al.^10^ indicated that the atogepant groups with doses of 10mg Once daily (OD), 30mg OD and 60mg OD showed a significant decrease in the number of MMDs, with mean change from baseline (CFB) in MMDs as -4 days, -3.8 days and -3.6 days respectively (p=<0.05). Similarly, the groups with doses of 30mg Twice daily (BID) and 60mg BID also demonstrated a significant decrease in MMDs, with values of -4.2 days and -4.1 days respectively (p=<0.05). The results of Ailani et al.^13^ study showed that atogepant in 10mg OD, 30mg OD, 60mg OD and Placebo doses had a significant decrease in the number of MMDs. The change was observed in the form of mean change from baseline in MMD as -3.7 days, -3.9 days, -4.2 days and -2.5 days respectively.

The study by Schwedt et al.^14^ reported that the atogepant groups with doses of 10mg OD, 30mg OD and 60mg OD showed a significant reduction in the number of monthly headache days during the period of weeks 9 to 12, with Least Squares (LS) mean changes from baseline of -4.2, -4.3 and -4.4 respectively (p=<0.001).

The results of the study by Goadsby et al.^10^ showed that the least squares mean difference in Monthly Headache Days (MHDs) was -2.9 days in the placebo group, -4.3 days in the 10mg OD atogepant group, -4.2 days in the 30mg OD atogepant group, -3.9 days in the 60mg OD atogepant group, -4.2 days in the 30mg BID atogepant group, and -4.3 days in the 60mg BID atogepant group. The mean change from baseline in MHDs in the study by Ailani et al.^133^ was -3.9 days in the 10mg OD group, -4 days in the 30mg OD group, -4.2 days in the 60mg OD group, and -2.5 days in the placebo group. In the study by Schwedt et al.^14^, the least squares mean difference in MHDs was -1.4 days in the placebo group and -3.2 days, -3.4 days, and -3.8 days in the 10mg OD, 30mg OD, and 60mg OD atogepant groups respectively, during the first 4 weeks. According to Ashina et al.^17^ 84.2% of participants who received 60mg OD of atogepant showed a decrease of 50% or more in their monthly migraine days after a 12-week treatment period.

Additionally, the results showed a maximum decrease in the number of monthly migraine days for patients taking 30mg BID or 60mg BID doses of Atogepant compared to the placebo group. The studies conducted by Ankrom et al.^9^, Min et al.^11^, Boinpally et al.^12^, Lipton et al.^15^, and Klein et al.^16^ did not present the efficacy of any doses of atogepant. However, these studies reported various treatment-related adverse events (TRAEs), which were essential to evaluate the safety of atogepant. Thus, these studies were included in this systematic review and are presented in Table 2, Table 3, Table 4, Table 5, Table 6, Table 7, Table 8, Table 9.

**Table 2:** Outcomes in Major Clinical Trials for Atogepant OD: Once daily, BID: Twice daily, SD - Single Dose, CFB change from baseline, MMDs monthly migraine days, MHDs monthly headache days, TRAEs treatment-related adverse events, SAEs serious adverse events, OR odds ratio, CI confidence interval*p <0.05, **p<0.001, vs. placebo; ^a^The data are presented as difference from Placebo

Study	Doses	CFB in MMDs after 12 weeks (95% CI)a	CFB in MHDs after 12 weeks (95% CI)a	≥ 50% reduction in MMDs (%) (OR, 95% CI)	CFB in acute medication use days (95% CI)a	Incidence of TRAEs (%)	Incidence of SAEs (%)	Drug discontinuation rate (%)
Ankrom et al.^9^	60 mg OD	-	-	-	-	23.3	0	15.3
Goadsby et al.^10^	10 mg OD	− 1.2* (− 1.9 to − 0.4)	− 1.4* (− 2.2 to – 0.5)	1.5 (1 to 2.3)	− 1.3 (− 2.0 to − 0.6)	18	0	4
	30 mg OD	− 0.9* (− 1.6 to − 0.3)	− 1.2* (− 1.9 to − 0.6	1.5 (1 to 2.1)	− 1.4 (− 2.0 to − 0.9)	21	0	6
	60 mg OD	− 0.7* (− 1.4 to − 0.1)	− 0.9* (− 1.6 to − 0.2)	1.4 (1 to 2)	− 1.1 (− 1.7 to − 0.5)	23	0	3
	30 mg BID	− 1.4* (− 2.2 to − 0.6)	− 1.3* (− 2.2 to − 0.4)	1.8* (1.2 to 2.9)	− 1.4* (− 2.1 to − 0.6)	21	0	6
	60 mg BID	− 1.3* (− 2.1 to − 0.5)	− 1.4* (− 2.3 to − 0.5)	2.0* (1.3 to 3.2)	− 1.2* (− 1.9 to − 0.5)	26	0	8
	Placebo	-	-	-	-	16	0	3
Min et al.^11^	170 mg OD	-	-	-	-	87	0	8.7
	Placebo	-	-	-	-	72.7	0	0
Boinpally et al.^12^	300 mg SD	-	-	-	-	1.7	0	0
	Placebo	-	-	-	-	-	-	-
Ailani et al.^13^, Schwedt et al.^14^, and Lipton et al.^15^	10 mg OD	− 1.2** (− 1.8 to − 0.6)	− 1.4** (− 2.0 to − 0.8)	3.1** (2 to 4.6)	− 1.3** (− 1.8 to − 0.8)	23.1	0.5	4.1
	30 mg OD	− 1.9** (− 1.9 to –0.8)	− 1.5** (− 2.1 to− 1.1)	3.5** (2. 4 to 5.3)	− 1.3** (− 1.8 to − 0.8)	14.9	0	1.8
	60 mg OD	− 1.7** (− 2.3 to − 1.2)	− 1.7** (− 2.3 to − 1.1)	3.8** (2.6 to 5.7)	− 1.5** (− 2.0 to − 1.0)	19.5	0	2.6
	Placebo	-	-	-	-	9	0	2.7
Klein et al.^16^	60 mg OD	-	-	-	-	8.8	3.4	25.4 (after open label treatment period) and 27.7 (at safety follow-up period)
Ashina et al.^17^	60 mg OD	-	-	84.2	-	18	4.4	31.6
	Placebo	-	-	-	-	36.2	3.6	31.2

**Table 3:** Adverse Events related to Gastrointestinal System SD - Single Dose, OD - Once Daily, BID - Twice Daily

Study	Constipation, n (%)	Nausea, n (%)	Gastroenteritis, n (%)	Increased alanine aminotransferase level, n (%)	Vomiting, n (%)	Diarrhea, n (%)	Abdominal discomfort, n (%)
Ankrom et al.^9^							
60 mg OD	3 (11.5)	0	-	-	-	3 (11.5)	-
Goadsby et al.^10^							
10 mg OD	1 (1)	3 (3)	-	-	-	-	-
30 mg OD	10 (5)	10 (5)	-	-	-	-	-
60 mg OD	8 (4)	11 (6)	-	-	-	-	-
30 mg BID	3 (3)	5 (6)	-	-	-	-	-
60 mg BID	4 (4)	8 (9)	-	-	-	-	-
Placebo	2 (1)	5 (3)	-	-	-	-	-
Min et al.^11^							
170 mg	2 (8.7)	3 (13.0)	-	-	-	2 (8.7)	2 (8.7)
Placebo	0	1 (9.1)	-	-	-	0	0
Boinpally et al.^12^							
300 mg SD	0	0	-	-	0	0	0
Placebo	1 (1.7)	0	-	-	0	1 (1.7)	0
Ailani et al.^13^							
10 mg OD	17 (7.7)	11 (5.0)	3 (1.4)	3 (1.4)	-	-	-
30 mg OD	16 (7.0)	10 (4.4)	2 (0.9)	2 (0.9)	-	-	-
60 mg OD	16 (6.9)	14 (6.1)	2 (0.9)	2 (0.9)	-	-	-
Placebo	1 (0.5)	4 (1.8)	6 (2.7)	6 (2.7)	-	-	-
Klein et al.^16^							
60 mg OD	23 (3.4)	23 (3.4)	15 (2.2)	4 (0.6)	14 (2.0)	-	-
Ashina et al.^17^							
60 mg OD	39 (7.2)	34 (6.3)	13 (2.4)	11 (2.0)	-	-	-

**Table 4:** Adverse Events related to Respiratory System OD - Once Daily, BID - Twice Daily

Study	Upper respiratory tract infection, n (%)	Naso-pharyngitis, n (%)	Sinusitis, n (%)	Influenza, n (%)	Coronavirus Infection, n (%)	Bronchitis, n (%)	Streptococcal Pharyngitis, n (%)
Ankrom et al.^9^							
60 mg OD	0	-	-	-	-	-	-
Goadsby et al.^10^							
10 mg OD	6 (6)	3 (3)	-	-	-	-	-
30 mg OD	14 (8)	11 (6)	-	-	-	-	-
60 mg OD	10 (5)	14 (8)	-	-	-	-	-
30 mg BID	6 (7)	1 (1)	-	-	-	-	-
60 mg BID	6 (7)	3 (3)	-	-	-	-	-
Placebo	15 (8)	4 (2)	-	-	-	-	-
Ailani et al.^13^							
10 mg OD	9 (4.1)	4 (1.8)	4 (1.8)	3 (1.4)	-	-	-
30 mg OD	13 (5.7)	8 (3.5)	3 (1.3)	2 (0.9)	-	-	-
60 mg OD	9 (3.9)	8 (3.5)	5 (2.2)	5 (2.2)	-	-	-
Placebo	10 (4.5)	8 (3.6)	3 (1.4)	2 (0.9)	-	-	-
Klein et al.^16^							
60 mg OD	38 (5.5)	33 (4.8)	25 (3.6)	17 (2.5)	15 (2.2)	14 (2.0)	-
Ashina et al.^17^							
60 mg OD	56 (10.3)	24 (4.4)	15 (2.8)	18 (3.3)	-	-	11 (2.0)

**Table 5:** Adverse Events related to Nervous System SD – Singel Dose, OD - Once Daily, BID - Twice Daily

Study	Somnolence, n (%)	Anxiety, n (%)	Dizziness, n (%)	Headache, n (%)
Ankrom et al.^9^				
60 mg OD	1 (3.8)	-	-	1 (3.8)
Goadsby et al.^10^				
10 mg OD	2 (2)	-	-	-
30 mg OD	2 (1)	-	-	-
60 mg OD	6 (3)	-	-	-
30 mg BID	1 (1)	-	-	-
60 mg BID	0	-	-	-
Placebo	2 (1)	-	-	-
Min et al.^11^				
170 mg	-	-	5 (21.7)	7 (30.4)
Placebo	-	-	1 (9.1)	3 (27.3)
Boinpally et al.^12^				
300 mg SD	0	-	0	0
Placebo	0	-	0	0
Ailani et al.^13^				
10 mg OD	7 (3.2)	2 (0.9)	-	-
30 mg OD	4 (1.8)	1 (0.4)	-	-
60 mg OD	4 (1.7)	5 (2.2)	-	-
Placebo	2 (0.9)	2 (0.9)	-	-
Klein et al.^16^				
60 mg OD	14 (2.0)	14 (2.0)	17 (2.5)	-
Ashina et al.^17^				
60 mg OD	-	16 (2.9)	17 (3.1)	-

**Table 6:** Adverse Events related to Musculoskeletal System OD - Once Daily, BID - Twice Daily

Study	Increased blood creatine kinase level, n (%)	Back Pain, n (%)	Arthralgia, n (%)	Neck Pain, n (%)	Musculoskeletal pain, n (%)	Muscle strain, n (%)
Ankrom et al.^9^						
60 mg OD	-	2 (7.7)	-	-	-	-
Goadsby et al.^10^						
10 mg OD	4 (4)	-	-	-	-	-
30 mg OD	3 (2)	-	-	-	-	-
60 mg OD	2 (1)	-	-	-	-	-
30 mg BID	6 (7)	-	-	-	-	-
60 mg BID	2 (2)	-	-	-	-	-
Placebo	3 (2)	-	-	-	-	-
Min et al.^11^						
170 mg	-	3 (13.0)	-	2 (8.7)	2 (8.7)	-
Placebo	-	0	-	0	0	-
Ailani et al.^13^						
10 mg OD	5 (2.3)	-	-	-	-	-
30 mg OD	2 (0.9)	-	-	-	-	-
60 mg OD	7 (3.0)	-	-	-	-	-
Placebo	2 (0.9)	-	-	-	-	-
Klein et al.^16^						
60 mg OD	-	17 (2.5)	15 (2.2)	-	-	-
Ashina et al.^17^						
60 mg OD	-	13 (2.4)	11 (2.0)	-	-	11 (2.0)

**Table 7:** Adverse Events related to Cardiovascular System OD - Once Daily

Study	Hypertensionn (%)	Vessel puncture site pain, n (%)	Hematoma n (%)
Min et al.^11^			
170 mg	-	3 (13.0)	2 (8.7)
Placebo	-	1 (9.1)	2 (18.2)
Ashina et al.^17^			
60 mg OD	14 (2.6)	-	-

**Table 8:** Adverse Events related to Urinary System OD - Once Daily, BID - Twice Daily

Study	Urinary tract infection, n (%)
Goadsby et al.^10^	
10 mg OD	2 (2)
30 mg OD	11 (6)
60 mg OD	5 (3)
30 mg BID	2 (2)
60 mg BID	3 (3)
Placebo	4 (2)
Ailani et al.^13^	
10 mg OD	3 (1.4)
30 mg OD	9 (3.9)
60 mg OD	9 (3.9)
Placebo	8 (3.6)
Klein et al.^16^	
60 mg OD	36 (5.3)
Ashina et al.^17^	
60 mg OD	28 (5.2)

**Table 9:** Miscellaneous Adverse Events SD – Single Dose; OD - Once Daily, BID - Twice Daily

Study	Fatigue, n (%)	Decreased appetite, n (%)	Erythema, n (%)	Feeling hot, n (%)	Chills, n (%)	Scratching, n (%)	Oro-pharyngeal Pain, n (%)	Weight Decrease, n (%)	Injury, poisoning, procedural complications, n (%)	Photophobia, n (%)
Ankrom et al.^9^										
60 mg OD	-	-	-	-	-	-	-	-	1 (3.8)	-
Goadsby et al.^10^										
10 mg OD	1 (1)	3 (3)	-	-	-	-	-	-	-	-
30 mg OD	2 (1)	3 (2)	-	-	-	-	-	-	-	-
60 mg OD	4 (2)	4 (2)	-	-	-	-	-	-	-	-
30 mg BID	1 (1)	0	-	-	-	-	-	-	-	-
60 mg BID	6 (7)	4 (4)	-	-	-	-	-	-	-	-
Placebo	4 (2)	1 (1)	-	-	-	-	-	-	-	-
Min et al.^11^										
170 mg	11 (47.8)	5 (21.7)	3 (13.0)	2 (8.7)	2 (8.7)	2 (8.7)	2 (8.7)	2 (8.7)	-	-
Placebo	4 (36.4)	0	1 (9.1)	0	0	1 (9.1)	2 (18.2)	0	-	-
Boinpally et al.^12^										
300 mg SD	-	-	-	-	-	-	-	-	0	0
Placebo	-	-	-	-	-	-	-	-	1 (1.7)	1 (1.7)
Ailani et al.^13^										
10 mg OD	3 (1.4)	-	-	-	-	-	-	-	-	-
30 mg OD	7 (3.1)	-	-	-	-	-	-	-	-	-
60 mg OD	9 (3.9)	-	-	-	-	-	-	-	-	-
Placebo	4 (1.8)	-	-	-	-	-	-	-	-	-
Klein et al.^16^										
60 mg OD	14 (2.0)	6 (0.9)	-	-	-	-	-	18 (2.6)	-	-
Ashina et al.^17^										
60 mg OD	14 (2.6)	-	-	-	-	-	-	14 (2.6)	-	-

Initially, participants were screened during a 4-week baseline period^110,13-15,17^. The treatment period for administering atogepant and placebo to participants and monitoring them in different studies varied, with duration of 4 weeks in^11^, 12 weeks in^10,13-15^, 40 weeks in^16^, and 52 weeks in^17^. The number of monthly migraine days (MMD), monthly headache days (MHD), and acute medication days were recorded during this time. The safety follow-up period also varied in duration, ranging from 4 weeks^10,13-17^ to 2, 4, and 8 weeks^11^. All data was measured through in-person visits. The data presenting all available changes from baseline in MMD, MHD, days of acute medication use, as well as ≥50% reduction in MMD, is provided in Table 2 for all included studies.

The incidence of adverse events was similar between the atogepant (22.7%) and placebo (28.6%) groups across all studies, with constipation (4.27% in 7 trials), nausea (4.77% in 7 trials), fatigue (8% in 5 trials), and somnolence (1.52% in 5 trials) being reported by most of the studies. However, 4 studies reported a significant number of patients with urinary tract infections as a result of treatment-related adverse events (TRAE), suggesting that future studies could include more thorough evaluation of the urinary system^10,13,16,17^.

Moreover, the incidence of serious TRAEs was low for both atogepant (0.58%) and placebo (0.9%) groups across all studies. Additionally, our analysis revealed that the gastrointestinal system was the most affected, while the cardiovascular system was the least affected. Treatment-related adverse events, migraines and have a high rate of side effects, which reduces their adherence. As a result, it is noteworthy that around 85% of subjects enrolled in atogepant clinical studies. In the current review, Atogepant is found to be according to body systems, are presented in Table 3, Table 4,Table 5, Table 6, Table 7, Table 8 , Table 9. The efficacy and safety of the drug were consistent across various subgroups, including gender, age, and duration of migraines. Overall, the results suggest that atogepant is an effective treatment option for reducing the frequency of migraines.

## 6. Discussion

Atogepant is a daily oral CGRP receptor antagonist that was recently licensed by the FDA for the prevention of episodic migraine. Earlier to CGRP therapy, non-migraine specific medications such as anticonvulsants, antidepressants, and cardiovascular medicines dominated migraine prevention. The older, non-CGRP medications are non-specific to migraines and have a high rate of side effects, which reduces their adherence. As a result, it is noteworthy that around 85% of subjects enrolled in atogepant clinical studies.

In the current review, Atogepant is found to be effective in migraine prophylaxis. We demonstrated a reduction in the frequency of migraines and headaches among the participants who received oral atogepant treatment compared to placebo. Atogepant improved the endpoints statistically significantly at all dosages. Additionally, there was minimal evidence of safety hazards. When the time course of effect of atogepant was investigated, it was discovered that there was a substantial reduction in MMDs and acute medication usage days from the first week of therapy, which was maintained all across the double-blind duration. These data suggest that atogepant has a quick and long-lasting impact^14^.

Atogepant's safety has been demonstrated in clinical trials, with the majority of TRAEs being minimal to moderate in severity.

The rate of TRAEs was higher with atogepant than with placebo, and it was related to dosage escalation. The serious adverse events (SAE) reported by Goadsby et al. included urethritis, cholecystitis, migraine, and significant depression; however, none of these were found to be related to atogepant^10^. An acute asthmatic attack, post-surgical laryngospasm, and optic neuritis were among the SAEs reported by Ailani et al^13^. Optic neuritis, a significant TRAE, was observed in the atogepant 10 mg group. The study found optic neuritis as an SAE with 10 mg of atogepant, which began on day 23 after randomization and resulted in reduced visual acuity. The SAE was found to be associated with atogepant. It resolved on its own, leaving no lasting vision impairment. Although the studies reported ≥ 3 times elevation in ALT/AST levels in some participants, no patient reported having a drug-induced liver injury by either study. Apart from the studies listed in the review, atogepant is currently being evaluated for long-term safety and tolerability. While clinical studies have demonstrated that atogepant is more effective than placebo in lowering MMD in persons with episodic migraine, no head-to-head evaluations of atogepant vs other migraine medicines have been conducted. Atogepant's role in migraines will most likely be similar to that of other CGRP medicines, as a secondary choice to less expensive oral therapies.

## 7. Conclusion

The novel oral medication for migraine prophylaxis, atogepant, appears to be safe and effective, having a quick and prolonged impact. Peers' effectiveness is often comparable. When taken in the prevention of migraine, atogepant is typically well tolerated, and no significant TEAEs or fatalities have been linked to its usage. As an oral medication, atogepant might be preferred by a considerable number of patients over injection-based treatments. Atogepant has the capability to enhance the quality of life for individuals suffering from migraines, and it is an important addition to the armamentarium of treatments for this common and debilitating condition. Several clinical studies are now underway to better explore both the potential and limitations of this novel medication.
